# Emergence of a Highly Virulent Porcine Epidemic Diarrhea Virus (PEDV) G2c Subtype in China: Isolation, Genetic and Pathogenic Characterization, and Cross‐Neutralizing Antibody Response

**DOI:** 10.1155/tbed/3811264

**Published:** 2026-02-17

**Authors:** Yang-Yang Li, Chuan-Hao Fan, Hai-Xia Li, Hui-Qiang Zhen, Ye-Qing Zhu, Shouyu Wang, Bin Wang, Yao-Wei Huang

**Affiliations:** ^1^ State Key Laboratory of Animal Disease Control and Prevention, South China Agricultural University, Guangzhou, China, scau.edu.cn; ^2^ College of Animal Science, Anhui Science and Technology University, Chuzhou, China, aust.edu.cn; ^3^ OptiX+ Laboratory, Wuxi University, Wuxi, China; ^4^ Guangdong Laboratory for Lingnan Modern Agriculture, College of Veterinary Medicine, South China Agricultural University, Guangzhou, China, scau.edu.cn; ^5^ Department of Veterinary Medicine, Zhejiang University, Hangzhou, China, zju.edu.cn

**Keywords:** cross-neutralizing response, G2c, pathogenicity, porcine epidemic diarrhea virus (PEDV), spike (S) protein mutations

## Abstract

Porcine epidemic diarrhea virus (PEDV), an enteropathogenic coronavirus causing high mortality in neonatal piglets, continues to threaten global swine industries. Frequent mutations in the spike (S) protein of PEDV, particularly in emerging variants, have substantially compromised commercial vaccine efficacy. Despite the emergence of G2c variants dominating recent epidemics, comprehensive studies integrating viral isolation, phylogenetics, structural modeling, cross‐neutralizing antibody response, and pathogenicity assessment remain insufficient. In this study, we successfully isolated a G2c strain (AHCZ02) and obtained 69 S gene sequences from nine provinces during 2021–2024. Phylogenetic analysis identified G2c variants as predominant (69.57%, 48/69) in current outbreaks. Structural comparisons revealed four G2c‐specific substitutions (N139D, I287M, F345L, and L998M) inducing conformational changes in critical S domains compared to G2a/G2b strains, potentially disrupting immune recognition. The results of serum neutralizing antibody (nAb) test using the AHCZ02 strain showed that G2c‐based feedback exposure strategies elicited 3.9‐fold higher geometric mean titers (GMTs) than S‐INDEL–based approaches. Furthermore, feedback exposure strategies of G2c (GMT = 480–1893) showed 12‐ to 189.3‐fold higher neutralizing activity versus conventional vaccines (GMT = 10–40). Pathogenicity assessment in neonatal piglets revealed 100% mortality within 66 h post‐AHCZ02 inoculation, accompanied by hallmark clinical manifestations including profuse watery diarrhea, rapid weight loss, and severe jejunal villus atrophy. Collectively, these findings provide evidence that G2c variants have developed S protein modifications associated with diminished vaccine efficacy, underscoring the need for next‐generation vaccines incorporating G2c‐specific antigenic determinants, and strengthened virological surveillance systems to effectively monitor and respond to PEDV evolutionary dynamics.

## 1. Introduction

Porcine epidemic diarrhea virus (PEDV), an enveloped, single‐stranded, and positive‐sense RNA virus of the genus *Alphacoronavirus* (family *Coronaviridae*), is the causative agent of porcine epidemic diarrhea (PED) [1, 2]. PEDV infection induces acute clinical symptoms including emesis, severe watery diarrhea, and dehydration, with mortality rates approaching 100% in neonatal piglets, posing significant economic risks to the global swine industry [3–5]. The 28 kb PEDV genome encodes 16 nonstructural proteins (NSP1‐16), four structural proteins: spike (S), envelope (E), membrane (M), nucleocapsid (N), and an accessory protein (ORF3). The S protein as the principal surface antigen contains dominant neutralizing epitopes and mediates viral attachment via receptor binding, facilitating host cell membrane fusion, and serving as the key target for vaccine‐induced immunity [6–9].

Under selective pressure, PEDV undergoes continuous variations, resulting in the emergence of distinct genotypes with substantial divergence in virulence and transmissibility [9–11]. Phylogenetic analysis of S gene classifies PEDV strains into three genotypes: classical G1 (prototype strains), epidemic G2 (variant strains), and recombinant S‐INDEL strains [2, 12, 13]. The G1 genotype further divides into G1a (CV777‐like) and G1b (DR13‐like) subtypes, the G2 genotype comprises three evolutionary branches: G2a (American epidemic strains, e.g., AH2012), G2b (2010–2020 Chinese isolates, e.g., AJ1102 and CH/SD2014), and the emerging G2c subtypes comprising recent Chinese variants (e.g., SD2021 and TJbc2023), and the S‐INDEL genotype evolves into two evolutionary branches: Clade 1 (e.g., OH851) and Clade 2 (e.g., AH2408) [14–18]. While G2 strains have traditionally been linked to hypervirulence, recent surveillance has detected attenuated G2a variants (e.g., CH/GXNN‐1/2018 and ZJ/2022) circulating in swine herds [19, 20]. The emerging G2c subtype has displaced previous variants as the dominant strain in Chinese swine populations [16, 21, 22], necessitating sustained monitoring of PEDV evolutionary dynamics.

The S protein of PEDV, comprising S1 and S2 subunits, is pivotal for viral pathogenesis. The S1 subunit mediates host receptor binding, while the S2 subunit contains fusion peptides (FPs) critical for membrane fusion [6, 23, 24]. As a highly immunogenic antigen, the S protein drives neutralizing antibody (nAb) production essential for vaccine‐mediated protection. At least five neutralizing epitopes have been characterized in the S protein: S1^0^ (aa 19–220) [25], S1^A^ (aa 435–485) [26], SS2/SS6 motifs in S1D (aa 748–755 and 764–771) [27], and the C‐terminal 2C10 epitope (aa 1368–1374) [28]. Systematic characterization of mutations in the S protein of emerging G2c variants, particularly within these functional domains, is imperative for rational vaccine design and elucidation of genetic diversity among circulating strains.

Vaccination remains the principal intervention for PED control. However, recurrent PEDV outbreaks persist across Chinese swine populations, including herds with rigorous vaccine protocols and high coverage rates [29, 30]. This trend indicates that commercial vaccines derived from G1a (CV777), G1b (ZJ08), and G2b (AJ1102) subtypes demonstrate declining cross‐protective efficacy against prevalent variants, particularly circulating G2c strains. Consequently, swine operations increasingly implement controlled feedback immunization strategies, and intentional viral exposure of gestating sows to farm‐specific pathogens enhances lactogenic immunity transfer, particularly in endemically affected operations [3, 31–33]. nAbs targeting the PEDV S protein demonstrate a strong correlation with protective immunity, feedback‐immunized herds sustain nAb levels exceeding clinical protection thresholds [34, 35]. A critical knowledge gap persists regarding the comparison of feedback‐induced versus vaccine‐mediated immunity against G2c variants, particularly in strain‐specific neutralization profiles.

In response to the emergence of genetically divergent PEDV variants evading existing immunity, this study comprehensively characterized circulating G2c subtype strains to elucidate their antigenic and pathogenic changes. Through an integrated approach combining epidemiological surveillance, viral isolation, phylogenetic analysis, structural modeling, cross‐neutralization assays, and pathogenesis assessment, we aimed to: (1) reveal the dominant PEDV strains in China; (2) characterize the signature amino acid mutations within the S glycoprotein of emergent G2c variants; (3) model the structural alterations of the S protein; (4) compare geometric mean titers (GMTs) in cross‐neutralization assays elicited by field exposure versus conventional vaccines; (5) evaluate the virulence of representative G2c subtype (AHCZ02 strain) in neonatal piglet challenge models. Our findings demonstrated that contemporary G2c subtype variants have acquired critical modifications in the S protein that facilitate evasion of vaccine‐induced immunity, underscoring the urgent need for updated vaccines targeting these emergent strains.

## 2. Materials and Methods

### 2.1. Ethical Declarations

All procedures complied with institutional requirements and local legislation and were approved by the Animal Experiment Ethics Committee of Anhui Science and Technology University (Approval Number 2025068).

### 2.2. Clinical Specimens, Sample Processing, and S Gene Sequencing

Rectal swab and intestinal tissue specimens were collected from 2021 to 2024 from pigs exhibiting acute diarrhea across nine Chinese provinces (Anhui, Guangxi, Hebei, Heilongjiang, Hubei, Jiangsu, Shandong, Shanxi, and Zhejiang; Supporting Information 1: Figure S1A). In a 2022 epidemiological survey, a commercial sow farm in Anhui province experienced an outbreak featuring watery diarrhea, vomiting, and 50% mortality, samples from this event were subsequently used for viral isolation. Samples were diluted with phosphate buffered saline (PBS), centrifuged at 3000 × *g* (4°C, 5 min), and supernatants were transferred to RNase‐free tubes. Viral RNA was extracted using the virus DNA/RNA Extraction Kit 2.0 (Vazyme, Cat. No: RM401), followed by cDNA synthesis with a reverse transcription kit (Vazyme, Cat. No: R323) according to the manufacturer’s instructions. Target genes (ORF3 and S) were amplified by PCR using 2 × Taq Master Mix (Vazyme, Cat. Number P112‐01) with published primers [36, 37], and the amplicons were sequenced by General Biosystems (Anhui) Co., Ltd. Finally, 68 complete S gene sequences were obtained and deposited in the National Center for Biotechnology Information (NCBI) GenBank database under accession numbers PV523936‐PV524003.

### 2.3. Cells and Virus Isolation

Vero E6 cells (ATCC CRL‐1586) were cultured in Dulbecco’s modified eagle medium (DMEM, Gibco, Cat. Number 11995065) supplemented with 10% fetal bovine serum (FBS, Gibco, Cat. No: 10270106), maintained at 37°C in a humidified 5% CO_2_ incubator. PEDV isolation was conducted following established protocols with slight modifications [38, 39]. Specifically, PEDV‐positive specimens were homogenized in PBS (10% *w*/*v*), centrifuged at 3000 × *g* for 15 min, and filtered through sequential 0.45 μm and 0.22 μm membranes. Confluent Vero E6 monolayers (≥ 80% confluence) were washed three times with PBS (1 mL per wash) and inoculated with 0.2 mL of filtered viral supernatant in maintenance medium (serum‐free DMEM supplemented with 0.3% Tryptose Broth [TPB, Solarbio, Cat. Number LA1660], 5 μg/mL trypsin [Sigma, Cat. Number T4799], and 100 U/mL penicillin–streptomycin [Beyotime, Cat. Number C0222]). Following 2 h of adsorption at 37°C with 5% CO_2_, maintenance medium replacement was performed. Cultures achieving 80% cytopathic effects (CPEs) underwent three freeze–thaw cycles (−80°C/37°C), with clarified supernatants (centrifuged at 3000 × *g* for 10 min at 4°C) either subjected to serial passage or stored at −80°C.

### 2.4. Indirect Immunofluorescence Assay (IFA)

Confluent Vero E6 monolayers cultured in six‐well plates were infected with the PEDV AHCZ02 strain at a multiplicity of infection (MOI) of 0.1. Following 24 h of incubation, infected cells were sequentially processed through fixation with 4% paraformaldehyde (PFA) for 20 min at room temperature (RT), permeabilization using 0.1% Triton X‐100 in PBS for 15 min at RT, and blocking with 5% bovine serum albumin (BSA) in PBS for 30 min at 37°C. Immunofluorescence staining involved primary antibody incubation with a mouse anti‐PEDV‐N monoclonal antibody (1:500, provided by Dr. Zhang Shuai, Yangzhou University) at 37°C for 60 min, followed by incubation with fluorescein isothiocyanate (FITC)–conjugated rabbit anti‐mouse IgG (H + L) secondary antibody (1:1000, Proteintech, Cat. No: SA00003‐1) at RT for 60 min. Nuclear counterstaining was performed with 4,6‐diamidino‐2‐phenylindole (DAPI, 1 μg/mL; Beyotime, Cat. Number C1002) for 15 min at RT. Fluorescence imaging was recorded using Nikon Ti2‐U inverted fluorescence microscope with a DS‐Qi2 camera system.

### 2.5. Viral One‐Step Growth Curve

Viral replication kinetics of the plaque‐purified PEDV AHCZ02 strain in Vero E6 cells were assessed via one‐step growth curve analysis. Viral titers were determined by median tissue culture infectious dose (TCID_50_/mL) following this protocol: virus was serially diluted in maintenance medium (tenfold gradient from 10^−1^ to 10^−10^) and inoculated into 96‐well cell monolayers (100 μL/well). At 48 h postinfection (hpi), CPE were monitored at 12‐h intervals until 120 hpi, with TCID_50_ calculated using the Reed–Muench method.

### 2.6. Next‐Generation Sequencing

Viral RNA was extracted from 300 μL nuclease‐treated tissue homogenates using the Virus DNA/RNA Extraction Kit 2.0 (Vazyme, Cat. Number RM401). Library preparation and Illumina sequencing were performed by Shanghai Tanpu Biotechnology Co., Ltd. (Shanghai, China). The workflow consisted of library construction with the NEB Next Ultra II RNA Library Prep Kit (NEB, Ipswich, MA, USA), adapter ligation followed by 10‐cycle PCR amplification for library enrichment, and equimolar pooling combined with denaturation and dilution to optimal sequencing concentration. Paired‐end sequencing (150 bp reads) was conducted on an Illumina NovaSeq 6000 platform (Illumina, San Diego, CA, USA). The complete PEDV AHCZ02 strain genome sequence is deposited in GenBank under accession number PQ682538.

### 2.7. Phylogenetic Analysis of the S Gene and Amino Acid Characteristics of the S Protein of PEDV

The reference sequences of the S gene and genome of PEDV strains were retrieved from the NCBI nucleotide database (https://www.ncbi.nlm.nih.gov/). In detailed, 91 complete genome and 167 S gene sequences (Supporting Information 2: Tables S1 and Supporting Information 3: Table S2, respectively) were selected for phylogenetic analysis. Maximum‐likelihood (ML) trees for both S gene and complete genome datasets were constructed using IQ‐TREE v2.2.0 [40], with optimal nucleotide substitution models selected through ModelFinder based on Bayesian Information Criterion (BIC) scores [41]. 1000 bootstraps were performed to ensure the confidence of the trees. Sequence alignment was performed with MAFFT v7.526 [42] followed by manual refinement, and final trees visualization were enhanced using the Interactive Tree of Life (iTOL) v6 platform (https://itol.embl.de/). The consensus amino acid of G1, G2a, G2b, G2c and S‐INDEL strains were identified by Jalview v2.11.4.1 (https://www.jalview.org/), individually. Then, the consensus amino acid of each subtype and the 69 sequences of this study were aligned and analyzed in Jalview v2.11.4.1 to reveal the amino acid differences among each subtype.

### 2.8. Molecular Modeling of the S Protein of PEDV

The three‐dimensional structures of the S protein for the PEDV subtypes (G2a, 2b, 2c, and AHCZ02) were generated by homology modeling using the automated mode of the SWISS‐MODEL server (https://swissmodel.expasy.org/). The Pintung 52 strain (PDB: 7W6M) was selected as the primary template for all subtypes. The global model quality of each generated model was evaluated using the built‐in QMEAN model. Structural alignments and annotations of each subtype were performed using the PyMOL v3.1.3 (https://pymol.org/) molecular visualization system.

### 2.9. Clinical Serum Sample Collection for Virus Neutralization Assays

Serum samples (*n* = 118) were collected from six commercial swine herds with documented feedback exposure practices and vaccination histories. These samples were categorized into six experimental groups based on feedback exposure and immunization strategy: Group 1, G2c subtype feedback exposure (PEDV GX02‐04‐CN strain, GenBank Accession Number PV523947) with inactivated vaccine booster (*n* = 24); Group 2, S‐INDEL subtype feedback exposure (PEDV AH08‐12‐CN strain, GenBank Accession Number PV523936) with inactivated vaccine booster (*n* = 25); Group 3, AJ1102 live‐attenuated vaccine prime with inactivated vaccine booster (*n* = 24); Group 4, prime‐boost regimen with inactivated vaccine only (*n* = 20); Group 5, CV777 live‐attenuated vaccine prime with inactivated vaccine booster (*n* = 20); Group 6, negative control, no PEDV exposure or vaccination (*n* = 5). All immunizations followed a standardized schedule: 14 days intervals between initial interventions (feedback exposure/live‐attenuated priming/inactivated priming) and booster doses (2 mL/dose). Serum collection occurred uniformly at 21 days postbooster. The inactivated vaccine formulation was uniformly based on the AJ1102 strain for all vaccinated groups. Notably, pigs in the vaccine group had no prior history of PEDV infection or vaccination before the study.

### 2.10. Virus Neutralization Assays

Neutralization assays were performed using an established protocol [34, 43] with minor modifications. Serum samples were heat‐inactivated at 56°C for 30 min and supplemented with the antibiotic solution. Serial twofold dilutions (1:10–1:10,240) were generated, with 250 μL of each dilution combined with an equal volume of PEDV inoculum (200 TCID_50_/0.1 mL). Following 1‐h incubation at 37°C, 100 μL of mixtures were transferred to Vero cell monolayers in 96‐well plates. After 1 h adsorption, cells were cultured in maintenance medium at 37°C for 3–5 days. CPE progression was monitored daily. The neutralizing titer (NT_50_) was defined as the reciprocal of the highest dilution capable of inhibiting 50% of the CPE. NT_50_ was calculated using the Reed–Muench method and analyzed using Student’s *t*‐distribution to calculate two‐sided 95% confidence intervals (95% CIs). Samples below the lower limit of detection for nAbs were imputed at a value of half the detection threshold prior to statistical analysis [44].

### 2.11. Experimental Infection in Piglets

Ten healthy 3‐day‐old male Landrace piglets (body weight: 1.00–1.20 kg) were sourced from a commercial farm with no history of PEDV vaccination and confirmed PED‐free status for 12 consecutive months. Prior to inclusion, piglets underwent a 24‐h acclimatization period in controlled environmental conditions (30 ± 1°C) at the Animal Experiment Center of Anhui Science and Technology University. During acclimatization, piglets exhibiting inability to stand or diarrhea were excluded. All piglets tested negative via qRT‐PCR for PEDV, porcine deltacoronavirus (PDCoV), rotavirus (PoRV), and transmissible gastroenteritis virus (TGEV). Piglets were fed sterile liquid milk replacer (20 mL/kg metabolic body weight) at 4‐h intervals. According to previous studies [15, 45], 10 piglets were randomly allocated to two groups (*n* = 5/group). The first five piglets were assigned to the control group (oral administration of 2 mL DMEM), and the remaining five to the PEDV challenge group (oral inoculum: 2 mL PEDV strain AHCZ02 [1 × 10^5^ TCID_50_/mL]). Both groups were housed in separate rooms maintained at a standardized temperature of 30 ± 1°C. Postinoculation, piglets were monitored at 6‐h intervals for clinical outcomes: body weight, survival, and fecal consistency scored as: 0 (formed), 1 (semiformed), 2 (liquid with particulates), or 3 (watery diarrhea). At the conclusion of the experiment (5 days postinoculation), all surviving pigs were humanely euthanized. Euthanasia was performed in accordance with the American Veterinary Medical Association (AVMA) Guidelines for the Euthanasia of Animals. Pigs were first sedated with telazol (4 mg/kg, intramuscular injection) to minimize stress and discomfort. Following effective sedation, a lethal overdose of sodium pentobarbital (150 mg/kg body weight) was administered intravenously. Death was confirmed by the absence of a palpable heartbeat, absence of respiratory movements, and loss of corneal reflex. Intestinal specimens were collected within 15 min of mortality and fixed in 4% PFA for histopathological evaluation.

### 2.12. Pathological Examination

Intestinal tissue samples (duodenum, jejunum, ileum, and colon) from both challenge and control groups were collected during necropsy. Tissues were fixed in 4% PFA at RT for 48 h, then subjected to standard histological processing involving dehydration, clearing, and paraffin embedding. Serial 5‐μm sections were hematoxylin–eosin (H&E) stained for morphological evaluation. Immunohistochemical (IHC) analysis of duodenal, jejunal, and ileal tissue sections were performed using a mouse anti‐PEDV‐N monoclonal antibody to assess viral antigen distribution in intestinal tissues.

### 2.13. Statistical Analysis

Statistical analyses were performed using GraphPad Prism v9.5 (La Jolla, CA, USA) and R v3.5.3 (R Foundation for Statistical Computing). nAb level data were analyzed using one‐way ANOVA and Student’s *t*‐test to calculate the nAb level of each sample and the GMT of each group with 95% CI. A threshold of  ^∗^ *p* < 0.05 was considered statistically significant, with more stringent levels denoted as  ^∗∗^ *p* < 0.01,  ^∗∗∗^ *p* < 0.001, and  ^∗∗∗∗^ *p* < 0.0001.

## 3. Results

### 3.1. Isolation and Identification of the PEDV AHCZ02 Strain

The PEDV AHCZ02 strain was obtained from a diarrheic neonatal piglet during a 2022 Anhui Province outbreak (Figure 1A). Three serial passages (P3) of PEDV AHCZ02 in Vero cells induced initial CPE, characterized by syncytium formation at 48 hpi (Supporting Information 1: Figure S1D). While P4 passage exhibited no detectable CPE, distinct syncytium reemerged at 48 hpi in P5 cultures. Subsequent passages (P6–P9) maintained consistent 48 hpi CPE initiation, with accelerated progression and enhanced cytopathic severity (Supporting Information 1: Figure S1D). To confirm PEDV infection, phase‐contrast microscopy and IFA were performed. At 24 hpi, microscopic analysis revealed extensive syncytium formation in AHCZ02‐infected monolayers (Figure 1B). Subsequent IFA using anti‐PEDV‐*N* monoclonal antibody with FITC–conjugated rabbit anti‐mouse IgG secondary antibody specifically detected cytoplasmic accumulation of viral nucleocapsid protein (Figure 1C). Furthermore, the one‐step growth kinetics of PEDV AHCZ02 showed a peak titer of 10^6.64^ TCID_50_/0.1 mL at 30 hpi (Figure 1D).

Figure 1Isolation and characterization of PEDV AHCZ02 strain. (A) The sample location of PEDV AHCZ02 strain was colored with yellow. The base map was from the ministry of natural resources of China (http://bzdt.ch.mnr.gov.cn/). Map Approval Number: GS (2019)1822, and no modifications have been made to the map boundaries. (B) Cytopathic effects (CPE) in Vero E6 cells infected with PEDV AHCZ02 (P9) at 24 hpi. Infected cells exhibited multinucleated syncytia formation (white arrows), contrasting with intact monolayers in mock‐infected controls (scale bar = 100 µm). (C) Immunofluorescence detection of PEDV‐N protein (green) in infected Vero E6 cells, with DAPI counterstaining (blue; scale bar = 100 µm). (D) One‐step growth kinetics of PEDV AHCZ02 in Vero E6 cells. Peak viral titers (10^6.64^ TCID_50_/0.1mL) were achieved at 30 hpi. All data are represented as mean ± SD of three independent experiments.

**Figure:**
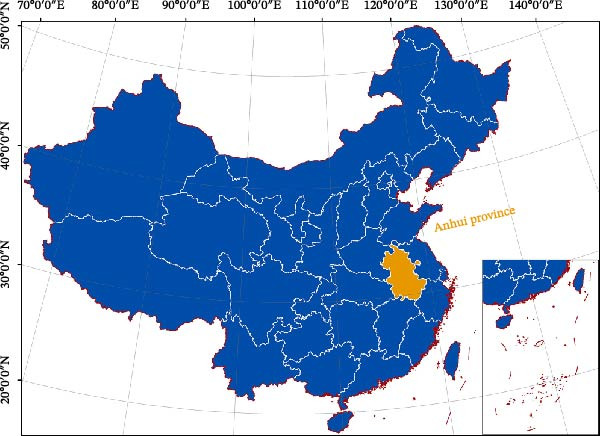
(A)

**Figure:**
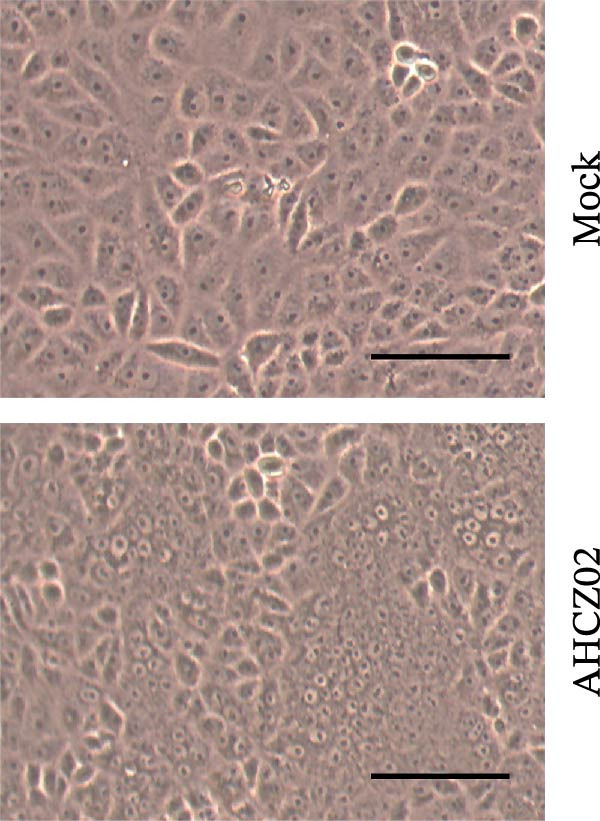
(B)

**Figure:**
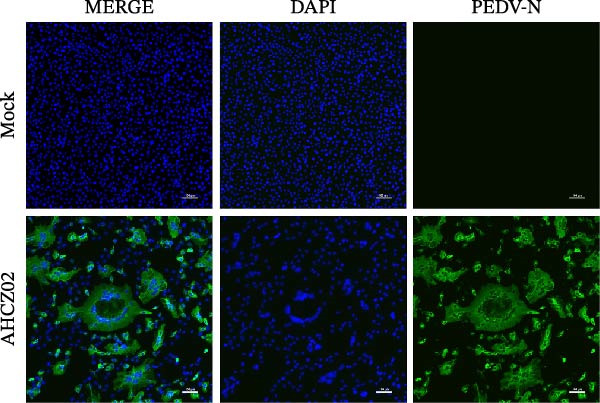
(C)

**Figure:**
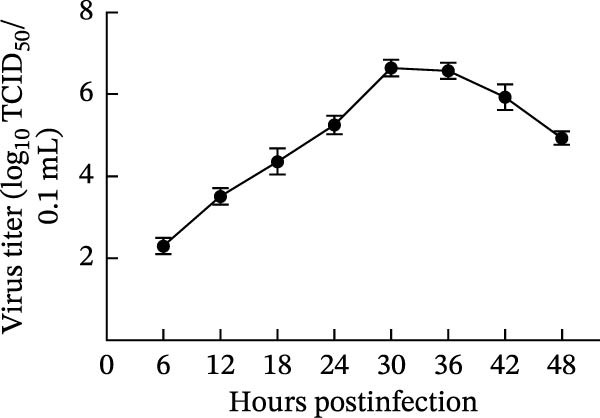
(D)

### 3.2. Epidemiology of PEDV From 2021 to 2024 in China

To investigate the molecular epidemiology of PEDV in China, rectal swab and intestinal tissue specimens were collected across nine provinces from 2021 to 2024 (Supporting Inforamtion 1: Figure S1A). PEDV‐positive specimens were identified through viral RNA extraction coupled with ORF3‐specific PCR amplification (Supporting Information 1: Figure S1B). Among the positive samples, 69 PEDV S gene were sequenced (Supporting Information 1: Figure S1C).

The ML tree shown that 69 Chinese strains belonged to G2 (92.75%, *n* = 64) and S‐INDEL variants (7.25%, *n* = 5). Furthermore, the G2 strains belonged into G2a (20.29%, *n* = 14), G2b (2.89%, *n* = 2), G2c (69.57%, *n* = 48) (Figure 2A), respectively. Notably, G2c strains accounted for 69.57% (48/69) of recent Chinese isolates (2021–2024), establishing this subtype as the current predominant PEDV strain.

Figure 2Phylogenetic analysis of PEDV AHCZ02 strain. Maximum likelihood (ML) trees of PEDV S gene (A) and complete genome (B). ML trees were constructed with IQ‐TREE (v2.20). Different subtypes were represented by different colors, strains sequenced in this study were labeled as normal red, and the AHCZ02 strain was labeled as bold.

**Figure:**
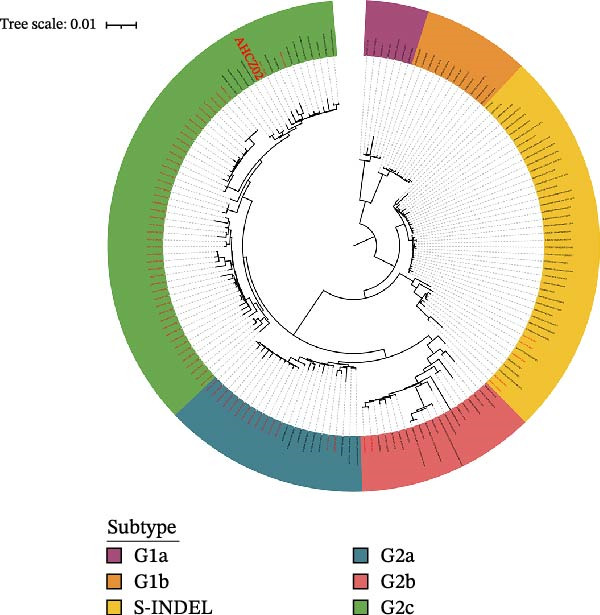
(A)

**Figure:**
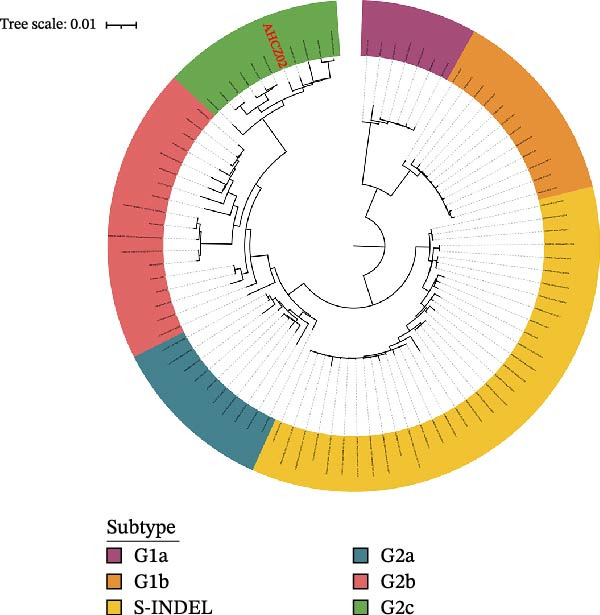
(B)

### 3.3. Genomic Characterization of the PEDV AHCZ02 Strain

Genomic analysis of the PEDV AHCZ02 strain (28,179 nt) revealed a canonical coronavirus genome architecture comprising seven conserved open reading frames (ORFs): ORF1a (12,309 nt), ORF1b (8,037 nt), S (4,158 nt), ORF3 (675 nt), E (231 nt), M (681 nt), and N (1,326 nt).

Whole‐genome phylogeny integrating AHCZ02 with global references confirmed its classification within G2c (Figure 2B). These data emphasize the necessity for continuous molecular surveillance to monitor emerging PEDV variants of epidemiological concern. Nucleotide identity comparisons showed that AHCZ02 exhibits maximal homology with G2c strains. Pairwise alignments revealed the following identity ranges relative to G2c references: ORF1ab (97.80–99.66%), S (97.72–99.43%), ORF3 (98.48%), E (99.03%–100%), M (99.56%–100%), and N (99.17–99.92%) genes (Supporting Information 4: Table S3), respectively. These findings confirm the genetic proximity of the PEDV AHCZ02 strain to the predominant G2c subtype.

### 3.4. Amino Acid Characteristics of the S Protein of PEDV

**Figure 3:**
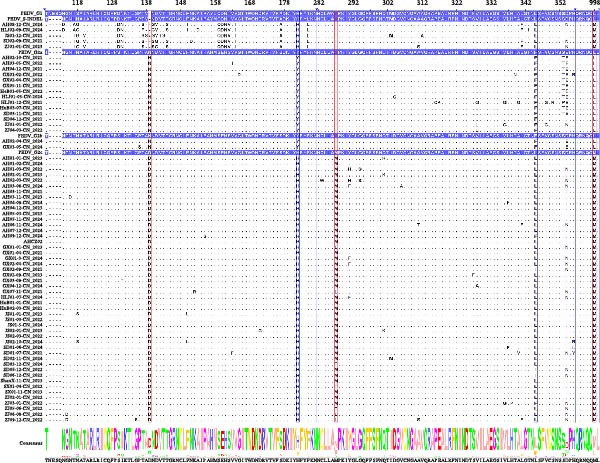
Sequence characterization of the S protein in PEDV G2c strains. The amino acid mutations of G2c subtype compared with other subtypes (G1, S‐INDEL, G2a, and G2b). The selected sequences including the domain sequences of each subtype and 69 S protein sequences obtained in this study. The sequences colored with blue represent the domain sequences of each subtype. Five amino acids were identified as mutations that different from other subtypes. The unique amino acid characteristics specific to G2c are represented in red box, those that share between G2c and S‐INDEL but distinct from other subtypes are in blue box.

Subtype‐specific consensus sequences of S gene were identified by comparing all S protein positions (Supporting Information 5: Table S4). Amino acid comparisons demonstrated that the PEDV AHCZ02 S protein share 99.06% amino acid identity with the G2c consensus profile, while containing five distinguishing substitutions relative to G2 a/G2b consensus sequences: N139D, Y182H, I287M, F345L, and L998M (Figure 3). Notably, G2a and G2b subtypes conserved ancestral residues (N139, Y182, I287, F345, and L998) at these positions, establishing these substitutions as molecular markers differentiating G2c variants. The S‐INDEL consensus sequence exhibited distinct residue patterns at position 139 (amino acid deletion), H182 (shared with G2c), I287 (divergent from G2c), L345 (shared with G2c), and L998 (shared with G2c; FIGURE 3). Three positions (H182, L345, and L998) showed identical residues between G2c and S‐INDEL subtypes, diverging from both G2a and G2b.

### 3.5. Analysis of the S Protein Structure

The PEDV S protein consists of two functional subunits (S1/S2) and 10 structural domains [45, 46]: signal peptide (SS), D0, N‐terminal domain (NTD), subdomain 1 (SD1), C‐terminal domain (CTD), subdomain 2 (SD2), FP, heptad repeat 1 (HR1), heptad repeat 2 (HR2), and transmembrane domain (TM). Proteolytic activation occurs at two critical cleavage sites: the S1/S2 junction and the S2’ site proximal to FP (Figure 4A). To assess structural impacts of mutations (N139D, Y182H, I287M, F345L, and L998M) across G2a, G2b, G2c, S‐INDEL subtypes, and AHCZ02 strain, comparative 3‐D models were generated based on AHCZ02 and subtype consensus sequences (Figure 4B,C). Structural modeling identified four mutations (N139D, I287M, F345L, and L998M) inducing localized conformational changes in AHCZ02 and G2c (Figure 4B,D): Site 1 (N139D): α‐helix formation (residues 116‐NTNATAR‐122) and β‐sheet shortening (residues 152‐KAIP‐155); Site 2 (I287M): β‐sheet shortening (residues 269‐VHGKVV‐274) and (residues 451‐IEVQ‐453); Site 3 (F345L): α‐helix elongation (residues 357‐LATF‐360) and β‐sheet disappears (residues 384‐VY‐385); Site 4 (L998M): α‐helix elongation (residues 1017‐VKEA‐1020).

Figure 4Structural homology modeling of the PEDV S protein highlighting critical molecular signatures. (A) Domain definitions of the PEDV AHCZ02 strain spike (S) protein. Annotated domains include: SS, signal sequence; D0, domain 0; NTD, N‐terminal domain of S1; SD1, subdomain 1 of S1; CTD, C‐terminal domain of S1; SD2, subdomain 2 of S1; FP, fusion peptide; HR1, heptad repeat 1; HR2, heptad repeat 2; TM, transmembrane domain. Proteolytic cleavage sites (S1/S2 and S2^′^) are indicated by red arrows. Unmodeled regions are denoted by dashed lines. (B) Structural localization of four critical molecular signatures (N139D, I287M, F345L, and L998M) in the PEDV AHCZ02 S protein predicted by SWISS‐MODEL. Mutations are shown as space‐filling models on the tertiary structure. (C) Spatial distribution of the four signature residues (red spheres) within the S protein 3D conformation. (D) Comparative structural analysis of the PEDV AHCZ02 S protein homology model (green) with G2a (cyan), G2b (lightmagenta), G2c (yellow), and S‐INDEL (orange) subtypes. Conformational conversion sites are marked with blue arrows and red arrows.

**Figure:**
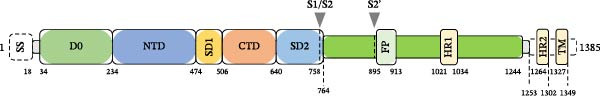
(A)

**Figure:**
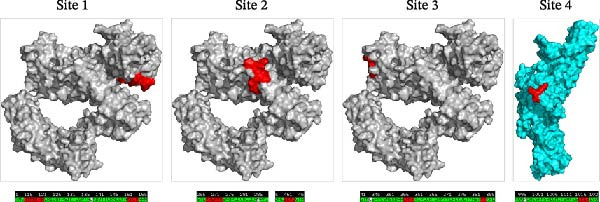
(B)

**Figure:**
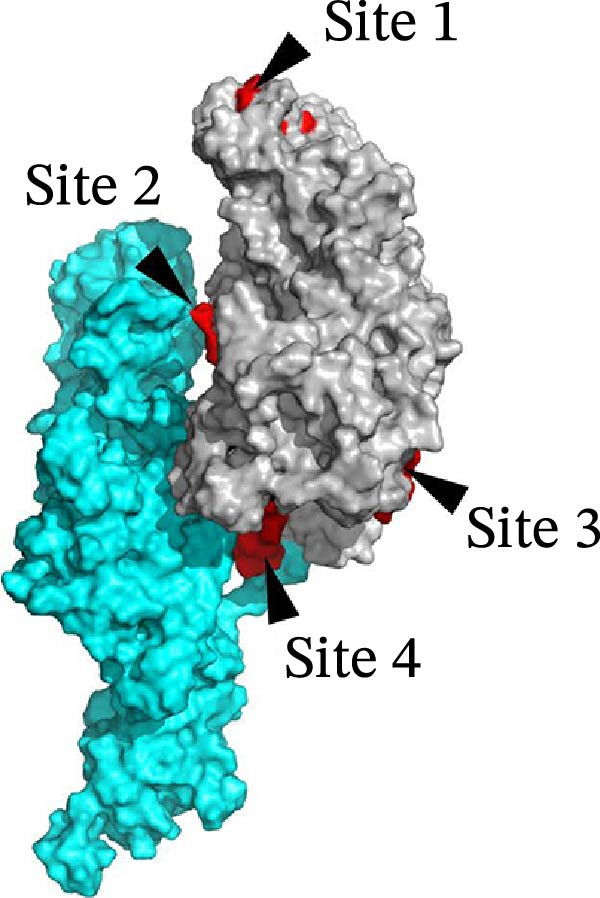
(C)

**Figure:**
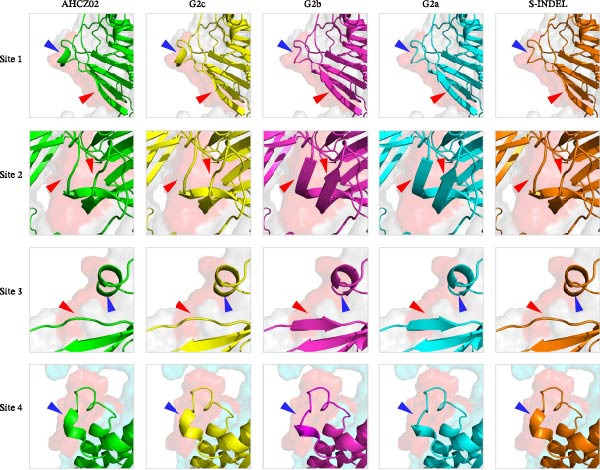
(D)

Notably, Sites 2 and 4 mapped to the S1/S2 interface in 3‐D models (Figure 4C). Comparative analyses revealed G2c subtype mutations predominantly induced secondary structural perturbations in adjacent regions rather than direct mutation site alterations.

### 3.6. Neutralization Efficacy Against Epidemiologically Dominant PEDV G2c Strains

**Figure 5:**
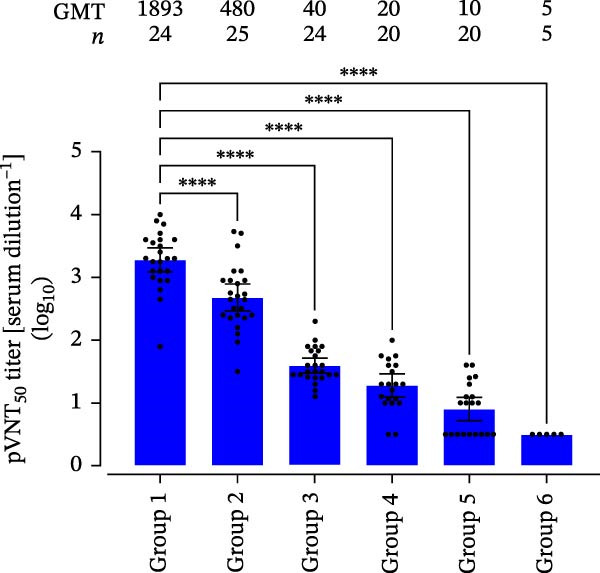
Neutralizing antibody titers in feedback‐exposed and vaccinated groups. Group 1 (G2c feedback and inactivated vaccine boost): geometric mean titer (GMT) = 1893 (95% CI: 1181–3200; *n* = 24); Group 2 (S‐INDEL feedback and inactivated vaccine boost): GMT = 480 (95% CI: 295–830; *n* = 25); Group 3 (AJ1102 live‐attenuated and inactivated vaccine boost): GMT = 40 (95% CI: 31–52; *n* = 24); Group 4 (inactivated vaccine prime‐boost): GMT = 20 (95% CI: 14–31; *n* = 20); Group 5 (CV777 live‐attenuated and inactivated boost): GMT = 10 (95% CI: 7–14; *n* = 20); Group 6 (no PEDV exposure and vaccination, *n* = 5): GMT = 5 (95% CI: 5; *n* = 5). The black data points correspond to the NT_50_ for individual biological replicates, while the blue illustrate the GMT with associated 95% confidence intervals (CI) depicted through vertical error bars. The black dots represent the neutralizing titer (NT_50_) of each sample, and the GMT of each groups were calculated, and the 95% CI is represented by the error bar ( ^∗∗∗^ *p* < 0.001,  ^∗∗∗∗^ *p* < 0.0001).

The epidemiological dominance of PEDV G2c strains in China implies potential limitations in current vaccine‐mediated immunity. Neutralization assays targeting G2c subtype AHCZ02 demonstrated substantial intergroup variation in GMTs among immunization groups stratified by immunization protocols and feedback‐exposure histories. In Group 1, G2c feedback exposure and inactivated vaccine boost elicited the highest neutralizing activity (GMT = 1,893; 95% CI: 1181–3200; *n* = 24); in Group 2, S‐INDEL feedback exposure and inactivated vaccine boost generated a GMT reduced to 480 (95% CI: 295–830; *n* = 25); in the vaccine‐only groups (Groups 3, 4, and 5), AJ1102 (G2b) live‐attenuated prime and inactivated boost yielded a GMT of 40 (95% CI: 31–52; *n* = 24), two‐dose inactivated vaccine prime‐boost regimens generated a GMT of 20 (95% CI: 14–31; *n* = 20), and CV777 (G1a) live‐attenuated prime and inactivated boost produced a GMT of 10 (95% CI: 7–14; *n* = 20); in nonimmunized controls (Group 6, *n* = 5), titers remained below the detection threshold (Figure 5). The G2c‐exposure group exhibited 3.9‐fold higher GMT than the S‐INDEL‐exposure group, confirming antigenic divergence between subtypes. Within vaccine‐only groups, AJ1102 (G2b)‐immunized pigs showed fourfold higher GMT compared to CV777 (G1a)‐immunized pigs, aligning with G2b’s closer genetic proximity to circulating G2c strains. Notably, the feedback‐exposed groups (Groups 1 and 2, GMT: 480–1,893) demonstrated 12‐ to 189.3‐fold higher neutralization than conventional vaccine regimens (Groups 3, 4 and 5, GMT: 10–40), highlighting current vaccines’ suboptimal efficacy against dominant G2c variants.

### 3.7. Pathogenicity Evaluation of PEDV AHCZ02 strain

**Figure 7:**
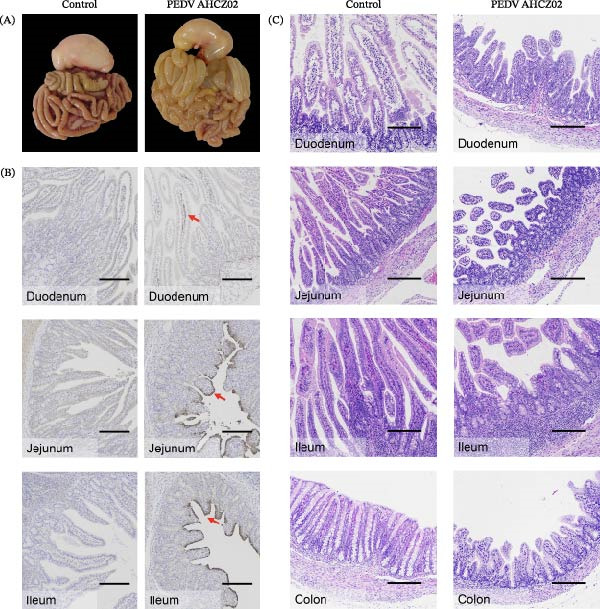
Pathogenicity assessment of PEDV AHCZ02 strain in piglets. (A) Gross lesions in PEDV AHCZ02‐infected piglets at necropsy. (B) Immunohistochemical (IHC) detection of PEDV antigens in intestinal tissues. Viral antigens (brown staining) are predominantly localized to jejunal and ileal villus epithelial cells (red arrows), with minimal detection in duodenum (red arrows; scale bars = 200 μm). (C) Histopathological changes in PEDV‐infected intestinal tissues hematoxylin and eosin (H&E) staining; scale bars = 200 μm.

Oral inoculation of 3‐day‐old piglets (*n* = 5) with 2 mL of PEDV AHCZ02 strain induced severe clinical‐pathological manifestations. All challenge group piglets (5/5, 100%) exhibited acute watery diarrhea (fecal score = 3) and emesis within 24 hpi (Figure 6A). Clinical progression manifested as systemic lethargy and dehydration, with initial mortality (1/5, 20%) occurring at 42 hpi and complete cohort lethality (5/5, 100%) by 66 hpi (Figure 6B). A >18% body weight reduction relative to baseline was recorded at 48 hpi (Figure 6C). Postmortem examination revealed severe intestinal pathology characterized by thin‐walled translucent small intestines, gas distension, and luminal accumulation of yellowish fluid (Figure 7A), contrasting with normal intestinal morphology in controls. Histopathological evaluation through H&E staining demonstrated significant villous atrophy across duodenal, jejunal, ileal, and colonic tissues (Figure 7C). IHC analysis confirmed PEDV nucleocapsid antigen mainly localization (brown staining) within jejunal/ileal villous enterocytes of infected piglets (Figure 7B), correlating with histomorphological alterations.

Figure 6Fecal scores, survival rates, and body weights between PEDV challenge and control group piglets. (A) Fecal scores in piglets. Clinical diarrhea severity was graded daily using a 0–3 scale (0 = normal, 1 = soft stool, 2 = loose stool, 3 = severe watery diarrhea). (B) Survival rates of piglets. (C) Body weight changes of piglets.

**Figure:**
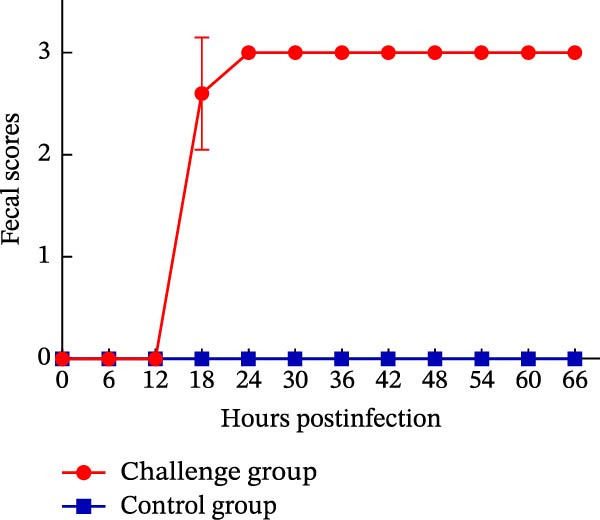
(A)

**Figure:**
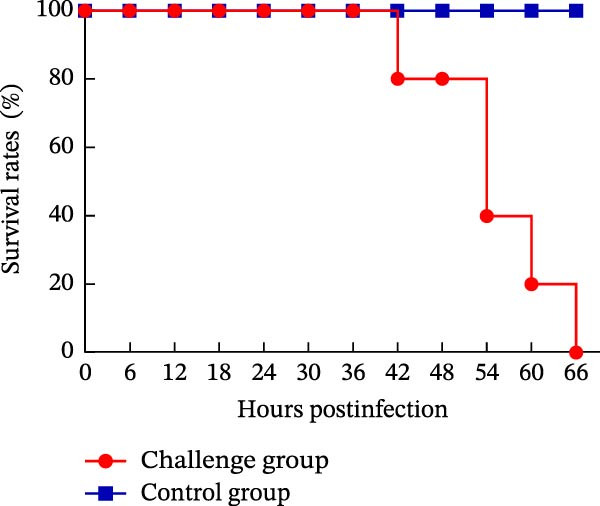
(B)

**Figure:**
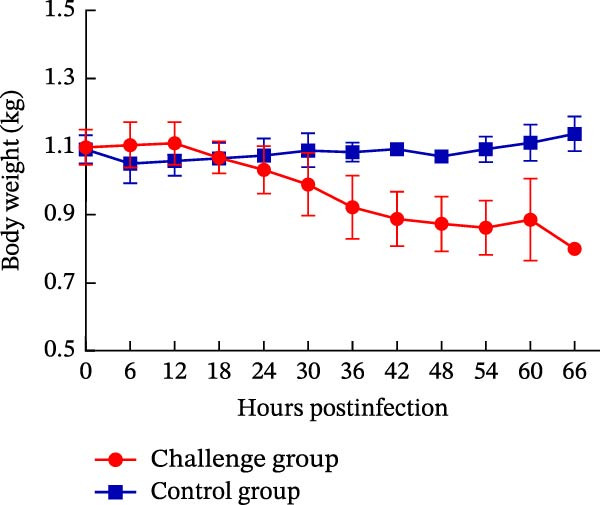
(C)

## 4. Discussion

Intinally identified in Europe, PEDV has evolved into a global threat to swine production, particularly the emergence of highly virulent G2 variants in China in 2010 [3, 47]. Despite widespread deployment of G1/G2‐based inactivated and live attenuated vaccines across Chinese swine populations, recurrent PED outbreaks with 50%–100% neonatal mortality persist in vaccinated herds [48]. Moreover, surviving piglets from such outbreaks, despite prior vaccination, frequently present with significant intestinal dysbiosis and severe, often irreversible, damage to the intestinal mucosa [49, 50]. Together, these observations indicate that current vaccination regimens fail to elicit sufficient cross‐protective immunity against the dominant circulating PEDV strains. Accumulating viral mutations may alter pathogenicity and antigenic profiles, potentially enabling immune evasion and complicating PED containment. Sustained surveillance of emerging variants is imperative to delineate genetic‐antigenic diversity for vaccine development and outbreak control. Therefore, in this study, an emergent G2c PEDV strain (AHCZ02) was isolated from diarrheic piglets in Anhui Province, China. Furthermore, comprehensive characterization analysis of the AHCZ02 strain including viral growth kinetics, S protein mutations, cross‐neutralization antibody response, and pathogenicity assessment were performed.

Viral titers, a key metric of replicative fitness and cellular adaptation, exhibit marked variation across PEDV genotypes. Comparative analyses demonstrate that G2 genotype strains consistently attain higher titers than S‐INDEL variants. Previous studies have reported the viral titers of different PEDV subtype, including HM2017 (P15:1.33 × 10^7^ TCID_50_/mL; [51]) and ZJ2022 (P10:10^6.725^ TCID_50_/mL; [19]) in G2a strains [19, 51], QIAP401 (P70 : 10^6.5^–10^7.0^ TCID_50_/mL; [52]) in G2b strains [52], TJbc2023(P6:10^6.455^ TCID_50_/mL; [19]), and HN‐1 (24 hpi: 8.18 × 10^7^ TCID_50_/mL; [53]) in G2c strains [53, 54], and EJS6 (10^3.75^ TCID_50_/mL; [13]) in S‐INDEL strains. In comparison with the above viral titers of each subtype, it’s revealed that G2 genotype strains consistently attain higher titers than S‐INDEL variants. Notably, the G2c subtype AHCZ02 strain demonstrated superior in vitro replication (10^7.64^ TCID_50_/mL), exceeding reported values for TJbc2023 and matching highly adapted variants like HN‐1. These findings suggest that enhanced replicative capacity of AHCZ02 may underpin its epidemiological dominance, identifying it as a promising vaccine candidate.

Surveillance data have identified G2c as the predominant subtype circulating in China. Multiple independent studies corroborate this epidemiological shift. For instance, Li et al. [22] reported 68.27% (71/104) of sequenced strains clustered into G2c during 2017–2021. Wang et al. [16] categorized 66.67% (20/30) of S genes as G2c (2017–2023). Peng et al. [21] observed a G2c prevalence of 87.28% (48/55) with co‐circulation of G2a (5.45%) and G2b (7.27%) subtypes. Phylogenetic analysis of 69 PEDV S genes (2021–2024) in this study revealed 69.57% (48/69) clustering within G2c which was in consistent with previous studies. Despite geographical and temporal disparities in surveillance, convergent evidence confirms G2c as the evolutionarily predominant genotype in China.

Accumulating viral mutations may alter pathogenicity and antigenic profiles, potentially enabling immune evasion and complicating PED containment. Prior studies have identified functional domains within the S protein: the S1^0^ region (aa 19–220) contains key neutralizing epitopes [25], the NTD interacts with 5‐N‐acetylneuraminic acid (a putative sugar co‐receptor) to facilitate epithelial cell entry [55, 56], and the S2 subunit mediates viral‐host membrane fusion [57]. In this study, five signature substitutions (N139D, Y182H, I287M, F345L, and L998M) distinguishing the G2c subtype (including the AHCZ02 strain) from G2a/G2b variants were identified. The N139D and I287M that distinguish G2c with other subtypes were the same as Li’s reported [16], but the Y182H, F345L, and L998M mutations were the first discovered in this study. 3‐D structural analyses revealed four mutations (N139D, I287M, F345L, and L998M) inducing distinct conformational changes: N139D (random coil→α‐helix transition and β‐sheet shortening within neutralizing epitopes); I287M (β‐sheet truncation in NTD); F345L (β‐sheet→random coil conversion and α‐helix elongation in NTD); L998M (α‐helix elongation in S2 subunit). These structural perturbations might explain the observed antigenic divergence. Notably, the N139D mutation is positioned in the S1^0^ neutralizing epitope, while F345L resides near the NTD co‐receptor binding interface, suggesting these mutations may play complementary roles in both evading immune responses and facilitating viral entry [58]. Therefore, mutations on N139D, I287M, F345L, and L998M may represent molecular signatures of the G2c subtype, and could be better to understand the evolution of PEDV G2c subtype.

The diminished nAb efficacy against current circulating PEDV strains are likely attributable to critical structural modifications in the S protein, which plays a central role in receptor recognition and viral entry. This observation is consistent with previous findings: Han et al. [15] reported that CV777‐based and AJ1102 polyclonal antibodies (PAbs) show diminished neutralization against epidemic strain PEDV WMB. Similarity, Xi et al. [59] found that immune sera from pigs immunized with G2b‐based vaccines exhibit negligible neutralizing activity against G2c strains, indicating significant antigenic divergence within neutralizing epitopes of G2c variants. nAb titers are a key correlate of immunogenic efficacy for PEDV vaccines. It has been established that maternal serum nAb titers (log_2_) of 1:377 to 1:774 or higher are associated with over 80% protection of piglets against PEDV infection [60]. In alignment with this protective threshold, our data demonstrate that feedback exposure strategies, particularly G2c feedback followed by inactivated vaccine boost (GMT = 1,893) and S‐INDEL feedback combined with inactivated vaccine boost (GMT = 480), elicited nAb levels exceeding the 1:377 benchmark. In contrast, conventional vaccination regimens resulted in markedly lower GMT values (10–40), falling well below the protective threshold. This substantial disparity in nAb induction may account for the superior effectiveness of feedback exposure strategies in PEDV prevention and control, as opposed to the limited protection afforded by conventional vaccination approaches.

Structural analyses identified four critical substitutions (N139D, I287M, F345L, and L998M) within key antigenic domains (S1^0^ region, NTD, and S2 subunit). These mutations might induce conformational changes in neutralizing epitopes, potentially explaining the substantial titer disparities between traditional vaccines and feedback‐induced immunity. Furthermore, the suboptimal nAb responses elicited by conventional vaccines, particularly against G2c variants, may account for the persistent circulation and epidemiological dominance of this subtype in Chinese swine populations. Therefore, these G2c‐specific S protein mutations could be better studied by the PEDV reverse genetics systems to establish a direct causal link between these mutations and hypervirulence.

The pathogenicity of PEDV AHCZ02 was confirmed in 3‐day‐old piglets. All inoculated piglets manifested acute watery diarrhea, emesis, and dehydration onset at 18 hpi, progressing to complete lethality by 66 hpi. This virulence profile corresponds with hypervirulent G2c strains (e.g., TJbc2023) [53]. Histopathological and IHC analyses demonstrated severe intestinal pathology predominantly localized in the jejunal and ileal regions, consistent with characteristic PEDV‐induced enteritis features, including villous atrophy and epithelial necrosis [61]. Moreover, limitations of this study including the exclusive use of 3‐day‐old piglets, potential biases from unblinded assessments, and restriction to a single PEDV strain (AHCZ02). Therefore, further investigation into the pathogenicity of PEDV G2c is required.

In conclusion, our epidemiological research revealed that G2c subgroup was predominant circulating in Chinese swine populations. And the AHCZ02 strain demonstrated high Vero E6 cell adaptation, characterized by five signatures mutations in the S glycoprotein and marked pathogenicity in 3‐day‐old piglets. Notably, current vaccination protocols yield diminished neutralization capacity against AHCZ02 in immunized herds. Our findings provide critical insights into the molecular features of prevalent G2c variants and emphasize the urgent need for developing strain‐matched vaccines.

## Funding

This work was supported by the National Key Research and Development Program of China (Grant 2025YFD1800902), the National Natural Science Foundation of China (Grants 32302854 and U22A20521), the Key Natural Science Research Project of Anhui Provincial Higher Education Institution (Grant 2023AH051840), the Talent Introduction Project of Anhui Science and Technology University (Grant DKYJ202205), the Veterinary Science Peak Discipline Project of Anhui Science and Technology University (Grant XK‐XJGF002), and the Guangdong Laboratory of Lingnan Modern Agriculture Project (Grant NG2022001).

## Conflicts of Interest

The authors declare no conflicts of interest.

## Supporting Information

Additional supporting information can be found online in the Supporting Information section.

## Supporting information


**Supporting Information 1** Figure S1: Epidemiological surveillance and CPE of PEDV AHCZ02 strain. (A) Geographical distribution of sampling location in this study. Nine sample locations were represented as different colors: AH (Anhui), GX (Guangxi), HeB (Hebei), HLJ (Heilongjiang), HuB (Hubei), JS (Jiangsu), SD (Shandong), SX (Shanxi), and ZJ (Zhejiang). The base map was from the ministry of natural resources of China (http://bzdt.ch.mnr.gov.cn/). Map Approval Number: GS (2019)1822, and no modifications have been made to the map boundaries. (B) ORF3‐specific PCR amplification of fecal samples. Lane M: DNA marker (DL 2000); Lane 1: ORF3‐positive sample (774 bp); Lane 2: negative control. (C) Full‐length S gene amplification verification. Lane M: DNA marker (DL 2000); Lanes 1–3: Representative S gene‐positive samples (1491–1695 bp); Lanes 4–6: negative controls. (D) Progressive cytopathic effects (CPE) during serial passage of PEDV AHCZ02 in Vero E6 cells: P3: Initial syncytium formation at 48 hpi; P5: syncytia reemer.

**Supporting Information 2** Table S1: 91 PEDV reference sequences to construct the complete genome phylogenetic tree.

**Supporting Information 3** Table S2: 167 PEDV reference sequences to construct the S gene phylogenetic tree.

**Supporting Information 4** Table S3: Nucleotide homology of PEDV AHCZ02 strain with G1, S‐INDEL, G2a, G2b, and G2c subtypes.

**Supporting Information 5** Table S4: Subtype‐specific consensus sequences of S gene of each subtype.

## Data Availability

All data supporting the findings of this study are included within the paper.
