# Supplementary material for: Emergence of a Highly Virulent Porcine Epidemic Diarrhea Virus (PEDV) G2c Subtype in China: Isolation, Genetic and Pathogenic Characterization, and Cross‐Neutralizing Antibody Response
Source: Transbound Emerg Dis. 2026 Feb 17;2026:3811264. doi: 10.1155/tbed/3811264 (PMC12910253; doi:10.1155/tbed/3811264)
Supplement: Supplementary file 1 — Supporting Information 1 Figure S1: Epidemiological surveillance and CPE of PEDV AHCZ02 strain. (A) Geographical distribution of sampling location in this study. Nine sample locations were represented as different colors: AH (Anhui), GX (Guangxi), HeB (Hebei), HLJ (Heilongjiang), HuB (Hubei), JS (Jiangsu), SD (Shandong), SX (Shanxi), and ZJ (Zhejiang). The base map was from the ministry of natural resources of China (http://bzdt.ch.mnr.gov.cn/). Map Approval Number: GS (2019)1822, and no modifications have been made to the map boundaries. (B) ORF3‐specific PCR amplification of fecal samples. Lane M: DNA marker (DL 2000); Lane 1: ORF3‐positive sample (774 bp); Lane 2: negative control. (C) Full‐length S gene amplification verification. Lane M: DNA marker (DL 2000); Lanes 1–3: Representative S gene‐positive samples (1491–1695 bp); Lanes 4–6: negative controls. (D) Progressive cytopathic effects (CPE) during serial passage of PEDV AHCZ02 in Vero E6 cells: P3: Initial syncytium formation at 48 hpi; P5: syncytia reemer. [file TBED-2026-3811264-s001.pdf]

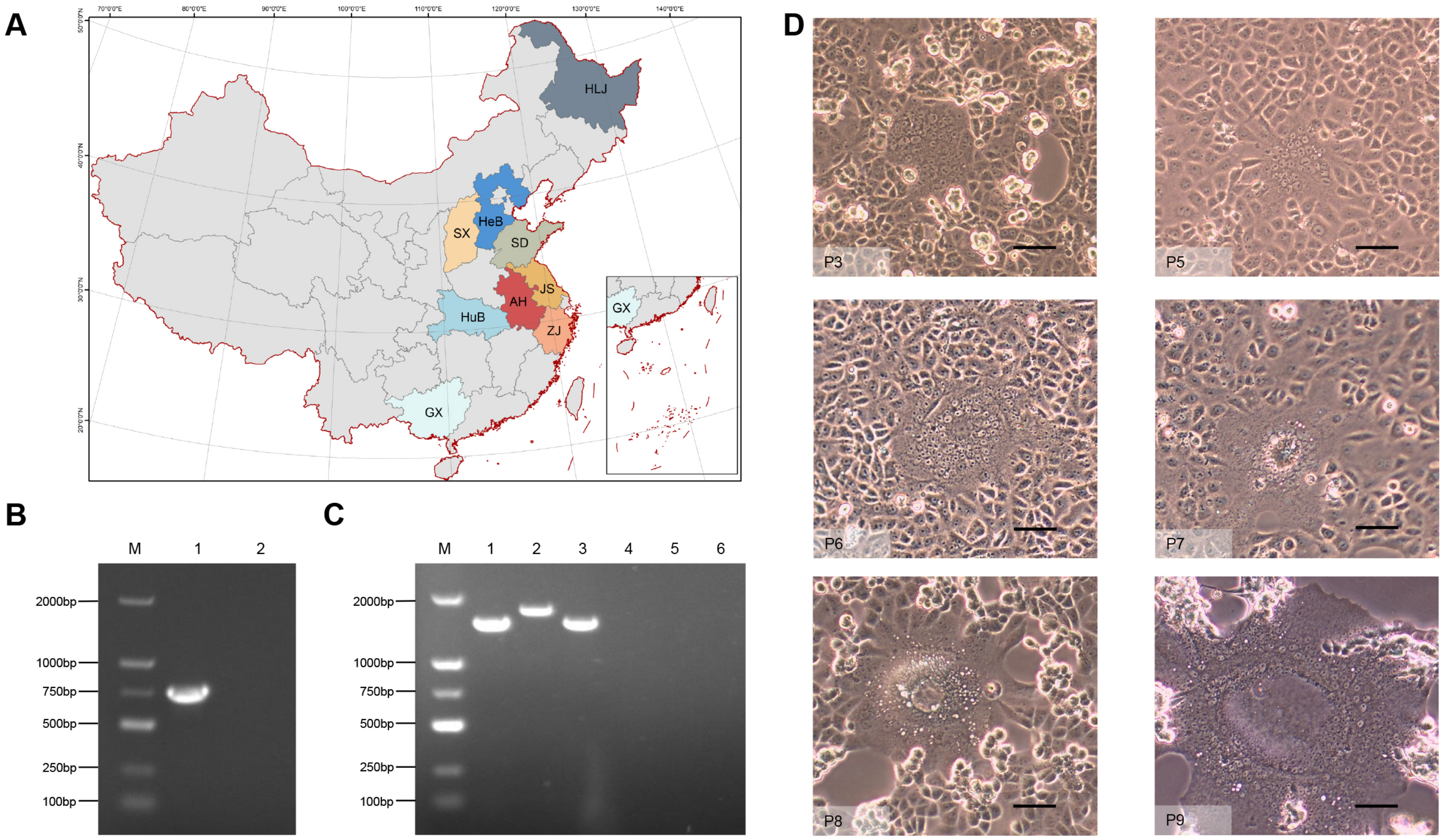

Figure S1. Epidemiological surveillance and CPE of PEDV AHCZ02 strain. (A) Geographical distribution of sampling location in this study. Nine sample locations were represented as different colors: AH (Anhui), GX (Guangxi), HeB (Hebei), HL J(Heilongjiang), HuB (Hubei), JS (Jiangsu), SD (Shandong), SX (Shanxi), ZJ (Zhejiang). The base map was from the ministry of natural resources of China (http://bzdt.ch.mnr.gov.cn/). Map Approval Number: GS (2019)1822. and no modifications have been made to the map boundaries. (B) ORF3-specific PCR amplification of fecal samples. Lane M: DNA Marker (DL 2000); Lane 1: ORF3-positive sample (774 bp); Lane 2: Negative control. (C) Full-length S gene amplification verification. Lane M: DNA Marker (DL 2000); Lanes 1-3: Representative S gene-positive samples (1491-1695 bp); Lanes 4-6: Negative controls. (D) Progressive cytopathic effects (CPE) during serial passage of PEDV AHCZ02 in Vero E6 cells: P3: Initial syncytium formation at 48 hpi; P5: Syncytia reemergence at 48 hpi; P6-P9: Accelerated CPE onset (48 hpi) with enhanced syncytium formation (scale bars = 100 µm; phase-contrast microscopy).
