# Supplementary material for: Emergence of a Highly Virulent Porcine Epidemic Diarrhea Virus (PEDV) G2c Subtype in China: Isolation, Genetic and Pathogenic Characterization, and Cross‐Neutralizing Antibody Response
Source: Transbound Emerg Dis. 2026 Feb 17;2026:3811264. doi: 10.1155/tbed/3811264 (PMC12910253; doi:10.1155/tbed/3811264)
Supplement: Supplementary file 4 — Supporting Information 4 Table S3: Nucleotide homology of PEDV AHCZ02 strain with G1, S‐INDEL, G2a, G2b, and G2c subtypes. [file TBED-2026-3811264-s004.docx]

**TABLE S3. Nucleotide homology of PEDV AHCZ02 strain with G1, S-INDEL, G2a, G2b and G2c subtypes.**

|  | Virus Gene | G1 subtype | S-INDEL subtype | G2a subtype | G2b subtype | G2c subtype |
| --- | --- | --- | --- | --- | --- | --- |
| AHCZ02 | ORF1ab gene | 96.53-97.64% | 97.7-98.01% | 97.78-98.11% | 97.64-98.00% | 97.8-99.66% |
|  | S | 92.97-94.14% | 95.27-96.07% | 97.25-98.14% | 96.78-98.12% | 97.72-99.43% |
|  | ORF3 | 89.39-95.45% | 90.91-92.42% | 92.42% | 92.42-98.48% | 98.48% |
|  | E | 96.62-97.58% | 98.55-99.03% | 99.03-99.52% | 98.07-100% | 99.03-100% |
|  | M | 98.09-98.97% | 98.09-98.83% | 98.38-98.68% | 98.24-99.85% | 99.56-100% |
|  | N | 94.72-96.91% | 96.76-97.44% | 96.91-97.21% | 96.08-98.11% | 99.17-99.92% |
